# The noble gas xenon provides protection and trophic stimulation to midbrain dopamine neurons

**DOI:** 10.1111/jnc.14041

**Published:** 2017-05-16

**Authors:** Jérémie Lavaur, Déborah Le Nogue, Marc Lemaire, Jan Pype, Géraldine Farjot, Etienne C. Hirsch, Patrick P. Michel

**Affiliations:** ^1^ Sorbonne Universités UPMC Univ Paris 06, Inserm, CNRS Institut du Cerveau et de la Moelle épinière (ICM) Hôpital Pitié‐Salpêtrière Paris France; ^2^ Air Liquide Santé International, Medical R&D Paris Saclay Research Center Jouy‐en Josas France

**Keywords:** astroglial cells, dopamine neurons, glutamate, neurodegeneration, NMDA, xenon

## Abstract

Despite its low chemical reactivity, the noble gas xenon possesses a remarkable spectrum of biological effects. In particular, xenon is a strong neuroprotectant in preclinical models of hypoxic‐ischemic brain injury. In this study, we wished to determine whether xenon retained its neuroprotective potential in experimental settings that model the progressive loss of midbrain dopamine (DA) neurons in Parkinson's disease. Using rat midbrain cultures, we established that xenon was partially protective for DA neurons through either direct or indirect effects on these neurons. So, when DA neurons were exposed to l‐*trans*‐pyrrolidine‐2,4‐dicarboxylic acid so as to increase ambient glutamate levels and generate slow and sustained excitotoxicity, the effect of xenon on DA neurons was direct. The vitamin E analog Trolox also partially rescued DA neurons in this setting and enhanced neuroprotection by xenon. However, in the situation where DA cell death was spontaneous, the protection of DA neurons by xenon appeared indirect as it occurred through the repression of a mechanism mediated by proliferating glial cells, presumably astrocytes and their precursor cells. Xenon also exerted trophic effects for DA neurons in this paradigm. The effects of xenon were mimicked and improved by the *N*‐methyl‐d‐aspartate glutamate receptor antagonist memantine and xenon itself appeared to work by antagonizing *N*‐methyl‐d‐aspartate receptors. Note that another noble gas argon could not reproduce xenon effects. Overall, present data indicate that xenon can provide protection and trophic support to DA neurons that are vulnerable in Parkinson's disease. This suggests that xenon might have some therapeutic value for this disorder.

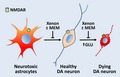

**Abbreviations used**
Ara‐C: cytosine β‐d‐arabinoside
DA: dopamine
DHR‐123: dihydro‐rhodamine‐123
DIV: days *in vitro*
FCS: fetal calf serum
GDNF: glial cell line‐derived neurotrophic factor
HS: horse serum
NMDA: *N*‐methyl‐d‐aspartate
PBS: Dulbecco's phosphate‐buffered saline
PDC: l‐*trans*‐pyrrolidine‐2,4‐dicarboxylic acid
PD: Parkinson's disease
ROS: reactive oxygen species
TH: tyrosine hydroxylase

Xenon is a monatomic gas that belongs to the family of noble gases or ‘inert gases’. Xenon's inertness results from the presence of a filled valence shell that prevents covalent bonds with other molecules under biologic conditions (Spaggiari *et al*. 2013). This lack of reactivity is somewhat relative as xenon can interact with biological components through non‐covalent forces (Liu *et al*. 2010; Sauguet *et al*. 2016). Such interactions are probably the basis of the interesting biological properties of xenon. For instance, xenon is an approved inhalation anesthetic with potent analgesic properties (Sanders *et al*. 2003; Banks *et al*. 2010). In addition to that, xenon has been reported to confer protection against acute neuronal damage in preclinical models of focal and global brain ischemia, spinal cord ischemia and traumatic brain injury (Ma *et al*. 2005; Banks *et al*. 2010; Yamamoto *et al*. 2010; Campos‐Pires *et al*. 2015). Neuroprotective effects have been generally observed in a range of concentrations that are comprised between 35 and 75% (Ma *et al*. 2005; David *et al*. 2008; Yang *et al*. 2012). It has been proposed that xenon may operate by activation of ATP‐sensitive potassium channels (Bantel *et al*. 2010) or of two‐pore potassium channels (Gruss *et al*. 2004). Yet, a majority of studies indicate that xenon is neuroprotective through antagonism of *N*‐methyl‐d‐aspartate (NMDA) glutamate receptors (Banks *et al*. 2010; Liu *et al*. 2010).

While beneficial effects of xenon have been largely reported under acute neurodegenerative conditions, it has been also suggested that the noble gas may also provide neuroprotection in situations where neuronal demise is more slowly progressing. In particular, we showed for the first time that a sustained exposure to xenon provides neuronal rescue in culture settings that are relevant to the pathogenesis of Alzheimer's disease (Lavaur *et al*. 2016a). Interestingly, we found that xenon also worked as a trophic factor toward basal forebrain cholinergic neurons, a neuronal population at risk in this disorder (Lavaur *et al*. 2016a,b).

These observations led us to explore the neuroprotective potential of xenon in experimental conditions that mimic neurodegeneration in Parkinson's disease (PD). To this aim, we used midbrain cultures and two experimental settings that model the progressive loss of dopamine (DA) neurons in this disorder. More specifically, when the cultures were exposed to the synthetic analog of glutamate l‐trans‐pyrrolidine‐2,4‐dicarboxylic acid (PDC) to generate sustained, low‐level excitotoxicity, we found that the ensuing loss of DA neurons was partially prevented if xenon replaced nitrogen in the cell culture atmosphere. In a situation where DA cell death was spontaneous and caused by a glial‐dependent mechanism involving astrocytes, xenon appeared to be protective through a repressive effect on these cells. Note that in addition to being neuroprotective, xenon also trophically stimulated DA neurons in this paradigm. The protective and trophic effects of xenon were improved by the NMDA receptor antagonist memantine and xenon itself seemed to operate by NMDA receptor antagonism.

## Material and methods

### Pharmacological reagents

PDC and memantine were obtained from Tocris (Lille, France) and Trolox from Sigma‐Aldrich (L'Isle d'Abeau Chesnes, France). Glial cell line–derived neurotrophic factor (GDNF) was from Eurobio AbCys (Courtaboeuf, France). The goat anti‐human/rat neutralizing GDNF antibody (AF‐212‐NA; RRID:AB_2111398) was purchased from R&D Systems Europe (Lille, France).

### Cell cultures protocols

Animals were housed, handled and taken care of in accordance with recommendations of the Guide for the Care and Use of Laboratory Animals of the National Institutes of Health (NIH Publication no. 85‐23, revised 1996) and the European Union Council Directives (2010/63/EU). Experimental procedures were authorized under number 0037 by the ethical committee on animal experiments Charles Darwin n. 5.

#### Midbrain cultures

Midbrain cultures were prepared using gestational day 15.5 embryos from Wistar female rats (Janvier LABS, Le Genest St Isle, France) that had been deeply anesthesized with sodium pentobarbital and killed by cervical dislocation. Once dissected, ventral midbrains were collected in Leibovitz L15 culture medium (Sigma Aldrich) and tissue pieces dissociated mechanically according to protocols described previously (Toulorge *et al*. 2011; Guerreiro *et al*. 2015). Dissociated cells in suspension were then seeded at a density of 1.2–1.5 × 10^5^ cells/cm^2^ onto Nunc 48 well multidish plates (ThermoFisher Scientific, Roskilde, Denmark) pre‐coated with 1 mg/mL polyethylenimine diluted in borate buffer, pH 8.3 (Sepulveda‐Diaz *et al*. 2016).

To study the protective potential of xenon for DA neurons in a situation of sustained, low‐level excitotoxic stress, we seeded and maintained midbrain cultures in Neurobasal medium (Gibco, Saint Aubin, France) supplemented with a B27 cocktail without antioxidants (Gibco), an N_2_ mix (Gibco), 2 mM glutamine and 100 IU/mL penicillin/streptomycin. Under these conditions, the cultures survived without the need for culture medium change until the end of the experimental protocol. Treatments with PDC were initiated at 12 days *in vitro* (DIV) and terminated at 16 DIV, except when indicated in the text.

We also used culture conditions, in which the death of DA neurons occurs spontaneously and progressively over a 2‐week period. For that, our cultures were maintained in minimum essential medium (Gibco) supplemented with 1 g/L glucose, 1 mM sodium pyruvate, 2 mM glutamine, and 100 IU/mL penicillin and streptomycin. The modified minimum essential medium also contained 10% horse serum (HS; Sigma‐Aldrich) and 10% fetal calf serum (FCS; Biowest LLC, Nuaillé, France, Les Ulis, France) during the initial phase of the culture, i.e., between 0 and 7 DIV. From 7 to 14 DIV, the concentrations of HS and FCS were reduced to 2% to ensure optimal preservation of the cultures (Wu *et al*. 2009). The exposure to gaseous atmospheres and/or conventional pharmacological treatments was initiated at 7 DIV, i.e., a stage where neurodegeneration is already ongoing. The cultures were generally processed at 14 DIV, except when specified in the text.

#### Astrocyte cultures

Astroglial cells in culture were obtained from the cortices of 15.5 day‐old rat Wistar embryos. Briefly, after dissection and trituration of brain tissue pieces in L15 Leibovitz medium, the cells in suspension were plated onto uncoated 75 cm^2^ Nunc culture flasks and maintained using modified minimum essential medium containing 10% HS and 10% FCS. After 7–8 days, of growth, the cells were recovered in Dulbecco's phosphate‐buffered saline (PBS) through mechanical scrapping to produce subcultures for functional studies. Sub‐cultured cells were plated onto PEI coated 48 wells and maintained in the same medium as before with HS and FCS reduced to 2%, only. The plating density was chosen so as to reach less than 50% confluency after 1–2 days of growth after plating. Then, gaseous and pharmacological treatments were applied for the next 3 DIV.

### Cell culture exposure to gases

After application of pharmacological treatments, 48‐well multi‐dish plates containing midbrain or astrocyte cultures were placed under humidified and hermetically sealed Plexiglas incubation chambers. The technical set‐up used for the supply of the pre‐defined gas atmospheres (20% O_2_, 5% CO_2_ and 75% of the test gas) has been previously described in detail (Lavaur *et al*. 2016a). The incubation chambers were maintained at 37°C in a conventional cell culture incubator for time periods indicated in the manuscript.

### Quantification of reactive oxygen species

Intracellular reactive oxygen species (ROS) were assessed using the cell membrane permeable probe dihydro‐rhodamine‐123 (DHR‐123) (#D23806; ThermoFisher Scientific, Waltham, MA, USA) according to a procedure described before (Rousseau *et al*. 2013). Measurements were made at a stage where ROS production is optimal in cultures exposed to PDC, that is 24 h after initiation of the treatment. For each culture condition, fluorescent images of 6 randomly chosen fields were acquired with a 20× fluorescence objective using a Nikon TE 2000 inverted microscope (Nikon, Champigny‐sur‐Marne, France) equipped with an ORCA‐ER digital camera and the HCimage Imaging software (Hamamatsu, Corp., Bridgewater, NJ, USA). Results were expressed in fractional change in fluorescence intensity relative to baseline in control cultures (*F*/*F*_0_) as described previously for other fluorescent probes (Toulorge *et al*. 2011; Sepulveda‐Diaz *et al*. 2016). The open source ImageJ software (Rasband 1997–2016) was used for quantification of the fluorescent signals. Note that because of the technical constraints imposed by our model system, we extrapolated ROS data from the whole population of neuronal cells to the few DA neurons present in midbrain cultures.

### Quantification of extracellular glutamate levels

For the detection of glutamate in the culture medium, we used the Amplex red glutamic acid/glutamate oxidase assay kit (#A12221) from ThermoFisher Scientific. The assay was performed according to the manufacturer's instructions using a fluorescence microplate reader Spectramax M4 (Molecular Devices, Sunnyvale, CA, USA) for quantitative assessment.

### Measurement of DA uptake

The functional integrity and synaptic function of DA neurons were evaluated by the ability of these neurons to accumulate [^3^H]‐DA by active transport (50 nM; 40 Ci/mmol; PerkinElmer, Courtaboeuf, France), as previously described (Rousseau *et al*. 2013).

### Incorporation of tritiated thymidine

Cell proliferation was assessed in mixed midbrain cultures and in astrocyte enriched cultures using the incorporation of tritiated‐thymidine as a marker of DNA synthesis as described previously (Mourlevat *et al*. 2003). Briefly, the cultures were exposed to thymidine [methyl‐^3^H] (1 μL/culture well, 70–90 Ci/mmol; Perkin Elmer) for 2 h using modified minimum essential medium without serum supplementation. After two rapid washes with cold PBS, the cells were recovered in distilled water for quantification of the radioactive signal by liquid scintillation spectrometry.

### Protein detection by immunofluorescence

Cultures fixed for 12 min using 4% formaldehyde in PBS, were then washed twice with PBS before an incubation step at 4°C for 24–72 h with a polyclonal anti‐tyrosine hydroxylase (TH) antibody diluted 1/1000 (T‐9237‐13; US Biologicals, Salem, MA, USA; RRID:AB_2650433) to detect DA neurons. We used a secondary IRDye 680RD antibody (LI‐COR Biosciences, Lincoln, NE, USA) and an Alexa fluor‐555 secondary antibody for near infrared fluorescence imaging detection and conventional fluorescence imaging detection, respectively.

### Image acquisition and cell quantification

In the PDC paradigm, the bottom surface area of each culture well was scanned in one piece using an LI‐COR Odyssey infrared imaging system (LI‐COR Biosciences) (Toulorge *et al*. 2011) and semi‐automatic quantification of DA cell numbers was made with the open source Icy software (de Chaumont *et al*. 2012). In the paradigm of spontaneous DA cell death, higher resolution composite images were generated with an Arrayscan XTi automated fluorescence microscope (ThermoScientific, Courtaboeuf, France), thus allowing both cell counting and morphometric analyses. Post‐hoc image analysis was then performed with the HCStudio software (Thermoscientific). With both methods, data analysis was performed over > 60% of the surface area of each culture well. For illustrative purposes, we also used digitized images of TH^+^ neurons that were taken with a Nikon Eclipse TE‐2000 microscope, equipped with an ORCA‐ER digital camera (Hamamatsu Photonics, Hamamatsu, Japan), operated with the HCI software (Hamamatsu).

### Statistical analyses

Data expressed as means ± SEM were analyzed, using the SigmaPlot 12.5 software (Systat Software Inc, San Jose, CA, USA). Each data point was derived from at least three independent experiments. In the PDC model, sets of data were assessed by one‐way analysis of variance followed the Student–Newman–Keuls *post hoc* test for all pairwise comparisons. In the spontaneous DA cell death model, data were analyzed with the Kruskal–Wallis one way analysis on ranks followed by the Student–Newman–Keuls *post hoc* test.

## Results

### Xenon provides partial rescue to DA neurons undergoing PDC‐induced degeneration

**Figure 1:**
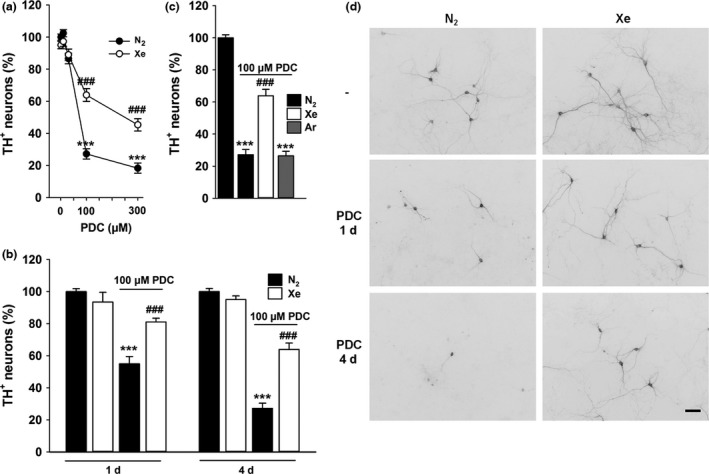
Xenon provides partial rescue against dopamine (DA) cell death induced by l‐*trans*‐pyrrolidine‐2,4‐dicarboxylic acid (PDC). (a) Survival rate of DA neurons (tyrosine hydroxylase, TH ^+^ cells) in cultures exposed or not to PDC (10–300 μM) for 4 days under an atmosphere containing 75% N_2_ or 75% Xe. Error bars indicate mean ± SEM (*n *=* *16). ***p <* *0.001 relative to control cultures and ^*###*^ *p *<* *0.001 relative to cultures receiving a same concentration of PDC under N_2_ atmosphere. (b) Survival of DA neurons exposed to 100 μM PDC for 1 (*n *=* *10) or 4 days (*n *=* *16) in an atmosphere containing 75% N_2_ or 75% Xe. Error bars indicate mean ± SEM. ***p <* *0.001 relative to age‐matched control cultures maintained under N_2_ atmosphere and ^*###*^ *p *<* *0.001 relative to age‐matched PDC‐treated cultures maintained under N_2_ atmosphere. (c) Survival rate of DA neurons in cultures exposed or not to PDC (100 μM) for 4 days under an atmosphere containing 75% N_2_, 75% Xe or 75% Ar. Error bars indicate mean ± SEM (*n *=* *16). ***p <* *0.001 relative to control cultures under N_2_ atmosphere and ^*###*^ *p *<* *0.001 relative to PDC‐treated cultures under N_2_ atmosphere. (d) Inverted fluorescence images illustrating the neuroprotective effects of 75% Xe for midbrain TH ^+^ neurons exposed for 1 or 4 days to 100 μM of PDC. Scale bar: 40 μm.

Midbrain cultures that had initially matured *in vitro* for 12 days in serum‐free conditions were exposed for the next 4 days to the synthetic analog of glutamate PDC to generate a slow and sustained excitotoxic process as it may occur in PD (Wallace *et al*. 2007). The survival of DA neurons (i.e., TH^+^ cells) was significantly affected at 100 μM of PDC and at concentrations above (Fig. 1a). At 100 μM PDC, we observed that the loss of TH^+^ neurons progressed from 45% to 73% at 1 and 4 DIV, respectively (Fig. 1b). Interestingly, PDC‐treated DA neurons were largely protected when the standard gas atmosphere containing 75% nitrogen was substituted with an atmosphere comprising 75% xenon. After 1 and 4 days of treatment with 100 μM PDC, the survival rate of TH^+^ neurons in cultures that were chronically exposed to xenon was 81% (vs. 55% in N_2_; *p *<* *0.001) and 64% (vs. 27% in N_2_; *p *<* *0.001), respectively (Fig. 1b). After 4 days of incubation with 300 μM PDC, i.e., treatment conditions where neuronal loss was more severe (> 80%), xenon was still neuroprotective but its efficacy to rescue DA neurons, more limited (Fig. 1a). The replacement of nitrogen by 75% argon, another noble gas having neuroprotective potential (Coburn *et al*. 2012; Zhao *et al*. 2016), failed to provide protection to TH^+^ neurons exposed to 100 μM PDC (Fig. 1c). An illustration of the protective effects of xenon for DA neurons is given in Fig. 1(d). We next aimed to further characterize the neuroprotective effects of xenon in the PDC paradigm.

### Xenon does not interfere with the presynaptic effect of PDC

**Figure 2:**
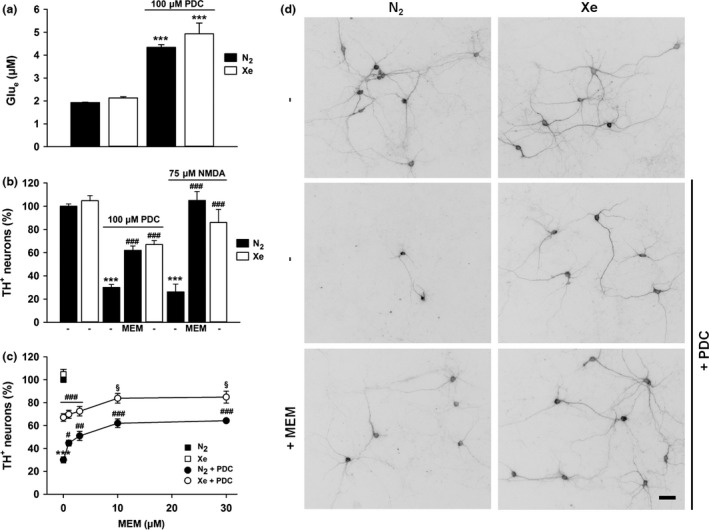
NMDA receptor antagonism accounts for the rescuing effect of xenon for l‐*trans*‐pyrrolidine‐2,4‐dicarboxylic acid (PDC)‐treated dopamine (DA) neurons. (a) Extracellular levels of glutamate measured in midbrain cultures treated or not with PDC for 24 h under gas atmospheres containing either 75% N_2_ or 75% Xe. Error bars indicate mean ± SEM (*n* = 9). ***p <* *0.001 relative to control cultures maintained under N_2_ atmosphere. (b) Survival rate of DA neurons exposed to PDC (100 μM) or NMDA (75 μM) for 4 days under an atmosphere containing either 75% N_2_ or 75% Xe. Comparison with a treatment with memantine (MEM, 10 μM) performed under a control N_2_ atmosphere. Error bars indicate mean ± SEM (*n = *6–9). ***p <* *0.001 relative to control cultures under N_2_ atmosphere and ^*###*^ *p *<* *0.001 relative to corresponding cultures exposed to either PDC or NMDA, under N_2_ atmosphere. (c) Survival of DA neurons exposed to PDC (100 μM) for 4 days in the presence, or not, of various concentrations of memantine (MEM, 1–30 μM) under gas atmospheres containing either 75% N_2_ or 75% Xe. Error bars indicate mean ± SEM (*n *=* *9). ***p <* *0.001 relative to control cultures under N_2_ atmosphere. ^*#*^ *p *<* *0.05, ^*##*^ *p *<* *0.01 and ^*###*^ *p *<* *0.001 relative to PDC‐treated cultures under N_2_ atmosphere. ^§^ *p *<* *0.05 relative to PDC‐treated cultures maintained under 75% Xe and to PDC‐treated cultures maintained under 75% N_2_ and a same concentration of memantine. (d) Inverted fluorescence images showing the impact that treatments with 75% xenon alone or 75% xenon + memantine (MEM, 10 μM) exert on the survival of DA neurons exposed to 100 μM PDC for 4 days. Scale bar 40 μm.

Because PDC operates through the blockade of glutamate uptake and stimulation of its release (Waagepetersen *et al*. 2001; Gouix *et al*. 2009; Lavaur *et al*. 2016a), we wished to estimate extracellular levels of the neurotransmitter in sets of cultures that had been exposed for 1 day to 100 μM PDC under a standard gas atmosphere containing 75% nitrogen. Our data show that the concentration of extracellular glutamate was increased by more than two‐fold (*p *<* *0.001 vs. control cultures under 75% N_2_) after 1 day of PDC treatment to reach ~ 4 μM, at this time point (Fig. 2a). The neurotransmitter remained elevated in cultures exposed to an atmosphere containing 75% of xenon (*p *<* *0.001 vs. control cultures under 75% N_2_), indicating that the noble gas was protective through a mechanism that was probably downstream to the presynaptic mechanism of action of PDC.

### NMDA receptor antagonism accounts for the rescue by xenon of PDC‐treated DA neurons

Xenon having the potential to operate by antagonism of NMDA receptors (Dickinson *et al*. 2007; Armstrong *et al*. 2012), we wished to compare its effect to that of memantine (10 μM), a non‐competitive antagonist of these receptors. Results show that after 4 days of incubation with 100 μM PDC, 10 μM memantine was about as effective as 75% xenon to protect DA neurons from degeneration (*p *<* *0.001 vs. control cultures under 75% N_2_) (Fig. 2b). Interestingly, xenon remained partially protective when NMDA (75 μM), the preferential agonist of NMDA receptors was used instead of PDC to kill DA neurons (*p *<* *0.001) (Fig. 2b). As expected, memantine was highly effective against NMDA‐induced DA cell death (*p *<* *0.001). This set of data supports the view that xenon operated itself by antagonism to NMDA receptors (Fig. 2b).

### Memantine reinforces the protective action of xenon for PDC‐treated DA neurons

As shown before, 75% xenon provided only partial protection to DA neuron exposed to 100 μM PDC. Therefore, we wished to determine whether the rescue provided by xenon could be improved by NMDA receptor antagonism with memantine. When used alone, memantine (1–30 μM) improved the survival of DA neurons in a concentration‐dependent manner with a plateau effect observed between 10 and 30 μM. The protective effect of 75% xenon against PDC was further improved (*p *<* *0.05) when memantine was used at 10 or 30 μM. Results describing the impact of combined treatments with xenon and memantine are described in Fig. 2(c) and (d).

### Xenon interferes with a death effector mechanism involving oxidative stress in PDC‐treated cultures

**Figure 3:**
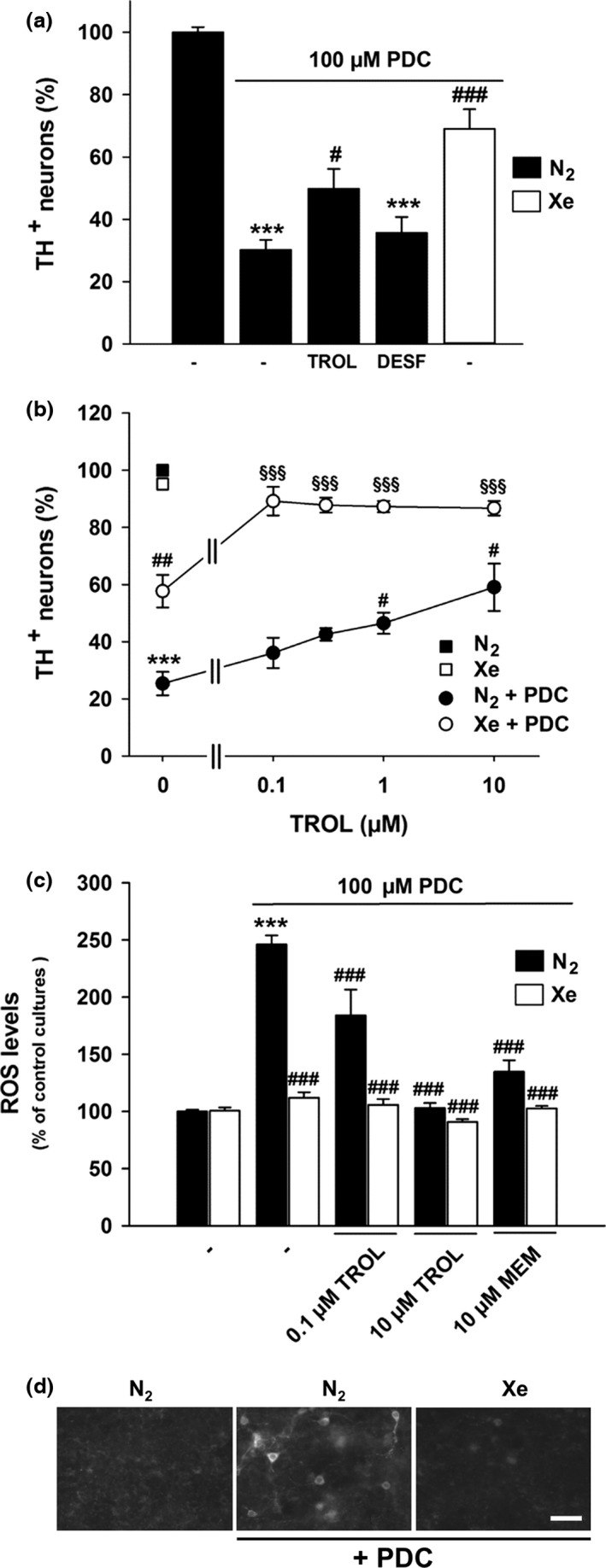
Xenon reduces the impact of oxidative insults in l‐*trans*‐pyrrolidine‐2,4‐dicarboxylic acid (PDC)‐treated dopamine (DA) neurons. (a) Survival of DA neurons in cultures exposed or not to PDC (100 μM) for 4 days and treated or not concomitantly with Trolox (TROL, 10 μM) or desferioxamine (DESF, 10 μM) under an atmosphere containing 75% N_2_. Comparison to cultures exposed to PDC under an atmosphere containing 75% Xe. Error bars indicate mean ± SEM (*n *=* *11–13). ***p <* *0.001 relative to control cultures under 75% N_2_ and ^#^ *p *<* *0.05 or ^###^ *p *<* *0.001 relative to PDC‐treated cultures maintained under 75% N_2_. (b) Survival of DA neurons exposed to PDC (100 μM) for 4 days in the presence or not of various concentrations of Trolox (0.1–10 μM) under gas atmospheres containing either 75% N_2_ or 75% Xe. Error bars indicate mean ± SEM (*n *=* *9). ***p <* *0.001 relative to control cultures under N_2_. ^#^ *p *<* *0.05 and ^##^ *p *<* *0.01 relative to PDC‐treated cultures under N_2_ atmosphere. ^§§§^ *p *<* *0.001 relative to PDC‐treated cultures maintained under 75% Xe and PDC‐treated cultures maintained under 75% N_2_ and a same concentration of Trolox. (c) Quantification of intracellular reactive oxygen species (ROS) production with the fluorescent probe dihydro‐rhodamine‐123 in midbrain cultures maintained under 75% N_2_ or 75% Xe and treated for 1 day with 100 μM PDC, alone or in combination with Trolox (TROL, 0.1 and 10 μM) or memantine (MEM, 10 μM). Error bars indicate mean ± SEM (*n *=* *6). ***p <* *0.001 relative to control cultures maintained under 75% N_2_ and, ^###^ *p *<* *0.001 relative to PDC‐treated cultures maintained under 75% N_2_. (d) Digitized images illustrating the impact of 75% Xe on ROS production in PDC‐treated cultures. Scale bar: 40 μm.

Oxidative stress being a component of PDC‐induced neuronal death (Nafia *et al*. 2008), we wished to compare the protective effect of xenon to that of two antioxidants, Trolox a cell‐permeable water‐soluble derivative of vitamin E and desferioxamine, a ferric iron chelator able to neutralize Fenton‐type reactions (Thomas *et al*. 2009). Even if less efficacious than xenon, Trolox (10 μM) provided substantial rescue to PDC‐treated DA neurons (*p *<* *0.05) whereas desferioxamine (10 μM) offered no protection at all Fig. 3(a).

In view of these results, we wished to determine whether Trolox had the capacity to improve xenon‐mediated protection against PDC. Surprisingly, Trolox which was not protective against PDC, at 0.1 and 0.3 μM, strongly reinforced the protective action that 75% xenon exerted onto DA neurons (*p *<* *0.001) Fig. 3(b). This suggested a synergistic action of the two treatments under these conditions. The effect of xenon was still improved (*p *<* *0.001) by higher concentrations of Trolox (1–10 μM) which were, however, partially protective on their own for DA neurons (Fig. 3b).

Additional studies performed with the ROS detection dye DHR‐123 revealed that oxidative stress returned to basal values in PDC‐treated neurons exposed to 75% xenon (*p *<* *0.001) (Fig. 3c and d). Despite having no protective effect *per se*, Trolox at 0.1 μM substantially reduced oxidative stress in PDC‐treated cultures. ROS returned to basal levels in the presence of 10 μM Trolox (*p *<* *0.001), i.e., a concentration having optimal protective effects for DA neurons. The blockade of NMDA receptors with a protective concentration of memantine (10 μM) also suppressed ROS produced by PDC (*p *<* *0.001). Note that none of the above treatments reduced ROS levels below control levels.

### Xenon is also protective for DA neurons that are spontaneously dying in serum‐supplemented midbrain cultures

**Figure 4:**
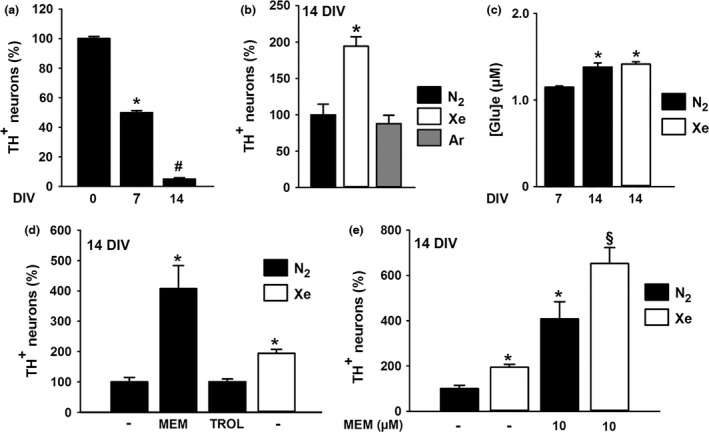
Xenon provides protection to spontaneously dying dopamine (DA) neurons through NMDA receptor antagonism. (a) Survival of DA neurons at days *in vitro* (DIV) 0, 7 and 14 in midbrain cultures supplemented with serum. Error bars indicate mean ± SEM (*n* = 6). *p <* *0.05 relative to control cultures after plating, i.e., at 0 DIV. ^#^ *p *<* *0.05 relative to 7 DIV control cultures. (b) Survival of DA neurons in cultures maintained between 7–14 DIV under an atmosphere containing 75% N_2_, 75% Xe or 75% Ar. Error bars indicate mean ± SEM (*n* = 9). *p <* *0.05 relative to control cultures maintained under N_2_ atmosphere. (c) Extracellular glutamate concentrations in 7 and 14 DIV midbrain cultures maintained under an atmosphere containing 75% N_2_ and in 14 DIV cultures kept under 75% Xe. Error bars indicate mean ± SEM (*n* = 9). *p <* *0.05 relative to 7 DIV cultures maintained under N_2_ atmosphere. (d) Survival of DA neurons treated between 7–14 DIV with Trolox (TROL, 10 μM), or memantine (MEM, 10 μM) and maintained under 75% N_2_. Comparison with cultures exposed to 75% Xe during the same time interval. Error bars indicate mean ± SEM (*n* = 9). *p <* *0.05 relative to control cultures maintained under 75% N_2_ atmosphere. (e) Survival of DA neurons maintained between 7–14 DIV under gas atmospheres containing either 75% N_2_ or 75% Xe in the presence or not of memantine (MEM, 10 μM). Error bars indicate mean ± SEM (*n* = 9). *p <* *0.05 relative to control cultures under 75% N_2_ atmosphere. ^§^ *p *<* *0.05 relative to cultures maintained under 75% Xe and cultures maintained under 75% N_2_ in the presence of memantine.

To further explore the neuroprotective potential of xenon, we used another experimental setting in which DA neurons maintained in serum‐supplemented culture medium degenerate spontaneously and progressively as a function of time. When monitoring DA cell numbers at different stages of the cultures, we established that about half of DA neurons were lost at 7 DIV and more than 95% by 14 DIV (Fig. 4a). Because we were particularly interested in determining whether DA cell rescue by xenon can occur when neurodegeneration is already well engaged, we started our treatments at 7 DIV and stopped them at 14 DIV. Our results show that xenon provided partial but significant (*p *<* *0.05) rescue to DA neurons in these conditions; the survival rate of TH^+^ neurons was increased by 194% in 14 DIV cultures chronically exposed to 75% of xenon. Note that exposing the cultures to an atmosphere containing 75% argon had no significant impact on DA cell survival in the same setting (Fig. 4b).

To determine whether xenon operated, here, like in the other setting by interfering with a slowly progressing excitotoxic process, we compared levels of the neurotransmitter in 7 and 14 DIV control cultures. Our results show that extracellular glutamate concentrations were low at 7 DIV and marginally increased at 14 DIV (1.15 vs. 1.4 μM), indicating that DA cell loss in this experimental context was probably unrelated to excitotoxicity. The presence of 75% xenon in the cell culture atmosphere also failed to modify glutamate levels in 14 DIV cultures (Fig. 4c).

### Xenon and memantine cooperate to protect DA neurons that are spontaneously dying in serum‐supplemented midbrain cultures

Even if the spontaneous loss of DA neurons was apparently not excitotoxic in nature, we found that TH^+^ cell numbers were increased by 407% (*p *<* *0.05) in the presence of 10 μM memantine in comparison to age‐matched control cultures. Trolox (10 μM) which was partially protective against PDC was, however, totally ineffective in this setting (Fig. 4d). Because treatments with either 75% xenon or 10 μM memantine provided partial rescue to spontaneously dying DA neurons, we tested whether a treatment combining xenon and memantine was able to further improve the survival of DA neurons. Our results clearly show that it is the case as the survival rate of DA neurons reached 652% of control cultures in these conditions (*p *<* *0.05 vs. each individual treatment) (Fig. 4e).

**Figure 5:**
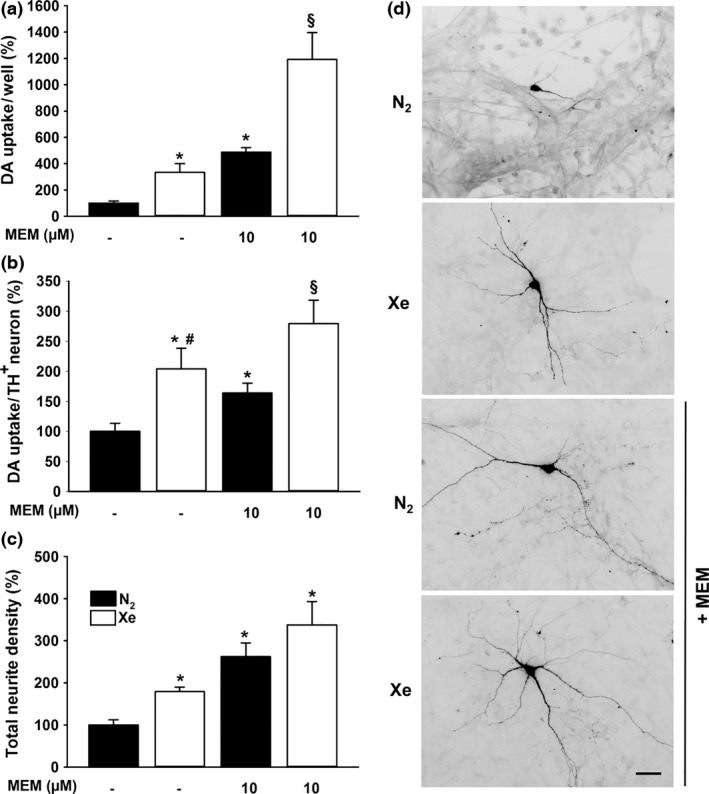
Xenon and memantine act cooperatively to improve the function and differentiation of spontaneously dying dopamine (DA) neurons. (a) Uptake of [^3^H]‐DA in 14 days *in vitro* (DIV) midbrain cultures treated for 7 days with or without memantine (MEM, 10 μM) under an atmosphere containing 75% N_2_ or 75% Xe. Data are means ± SEM (*n* = 9). *p <* *0.05 relative to control cultures under 75% N_2_. ^§^ *p *<* *0.05 relative to cultures maintained under 75% Xe and cultures maintained under 75% N_2_ in the presence of memantine. (b) DA uptake per DA (tyrosine hydroxylase, TH ^+^) neuron in 14 DIV midbrain cultures receiving the same treatments as in (a). Data are means ± SEM (*n* = 9). *p <* *0.05 relative to control cultures under N_2_ atmosphere. ^#^ *p *<* *0.05 relative to memantine‐treated cultures under N_2_ atmosphere. ^§^ *p *<* *0.05 relative to cultures maintained under 75% Xe and cultures maintained under 75% N_2_ in the presence of memantine. (c) Measurement of total neurite density per DA neuron in 14 DIV midbrain cultures receiving the same treatments as in (a) and (b). Data are means ± SEM (*n* = 9). *p <* *0.05 relative to control cultures under 75% N_2_ atmosphere. (d) Inverted fluorescence images representative of the impact that xenon and memantine have on the morphology of DA neurons when treatments are used, separately, or in combination. Scale bar 20 μm.

We next studied whether DA neurons rescued by xenon were functional. For that we assessed their ability to accumulate [^3^H]‐DA by active transport (Rousseau *et al*. 2013; Guerreiro *et al*. 2015). Coherent with the survival promoting effects of xenon for TH^+^ neurons, we found that a gas atmosphere enriched in xenon increased the uptake of DA/culture well by 333% in 14 DIV cultures (*p *<* *0.05) (Fig. 5a). Meanwhile, the uptake of DA was increased by 487% in midbrain cultures treated with 10 μM memantine under a control atmosphere containing 75% nitrogen (*p *<* *0.05). Of interest, performing the treatment with memantine under 75% xenon, further improved DA uptake, which reached in these conditions 1191% of control values (*p *<* *0.05 vs. each individual treatment). When the measure of DA uptake was expressed relative to TH^+^ cell numbers (Fig. 5b), the effect of 75% xenon on this parameter was greater than that of memantine (*p *<* *0.05) under 75% nitrogen (204% vs. 163%, respectively). Again, the effect of xenon appeared further enhanced by memantine (279%) (*p *<* *0.05). Altogether, these results show that xenon has the ability not only to preserve the survival of DA neurons but also to stimulate their function (Fig. 5b).

### Xenon exerts trophic effects on spontaneously dying DA neurons

DA uptake sites being preferentially located on neuritic extensions of DA neurons (Ciliax *et al*. 1995), we wished to determine whether the increase in DA uptake induced by xenon was partly because of the trophic action of the noble gas on these neurons. To address this question, we quantified the total density of TH^+^ neurites with respect to the number of DA neurons in 14 DIV midbrain cultures maintained under control or xenon‐enriched atmospheres in the presence or not of 10 μM memantine. Consistent with our hypothesis, the total neurite density/TH^+^ neuron was increased by 179% under 75% xenon (*p *<* *0.05). Besides, treatments with 10 μM memantine performed under gaseous atmospheres containing either 75% nitrogen or 75% xenon enhanced this parameter by 262% and 337%, respectively (Fig. 5c) (*p *<* *0.05). Figure 5(d) illustrates the impact of previous treatments on the morphology of DA neurons.

### GDNF is not involved in the effects of xenon

**Figure 6:**
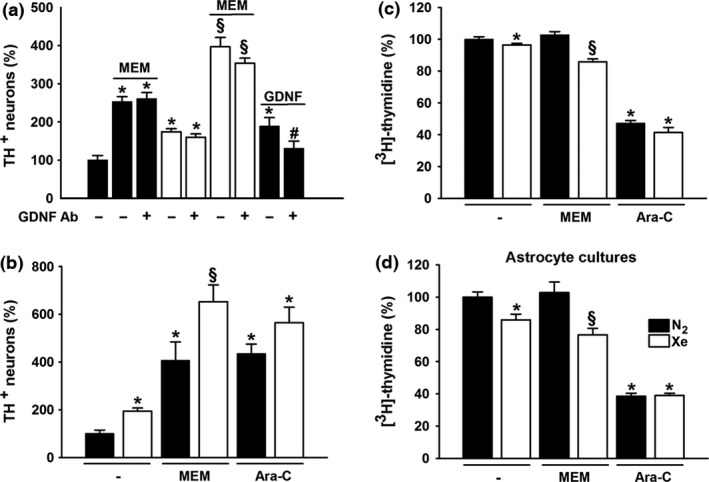
Xenon rescues spontaneously dying dopamine (DA) neurons through a repressive effect on astrocytes. (a) Survival of DA neurons in 14 days *in vitro* (DIV) midbrain cultures previously maintained for 7 days under 75% N_2_ or 75% Xe, in the presence or absence of memantine (MEM, 10 μM). Comparison with glial cell line‐derived neurotrophic factor (GDNF) (20 ng/mL) treatment in cultures kept under a control atmosphere. Impact of a GDNF neutralizing antibody (10 μg/mL) on all previous treatments. Data are means ± SEM (*n* = 9). *p <* *0.05 relative to control cultures under 75% N_2_. ^*#*^ *p *<* *0.05 relative to corresponding treatments without GDNF antibody_._^§^ *p *<* *0.05 relative to cultures maintained under 75% Xe and cultures maintained under 75% N_2_ in the presence of memantine. (b) Survival of DA neurons in 14 DIV midbrain cultures maintained under 75% N_2_ or 75% Xe for 7 days in the presence or absence of memantine (MEM, 10 μM) or cytosine β‐d arabinoside (Ara‐C) (10 μM). Data are means ± SEM (*n* = 9). *p <* *0.05 relative to control cultures under 75% N_2_. ^§^ *p *<* *0.05 relative to cultures maintained under 75% Xe and cultures maintained under 75% N_2_ and the same non‐gaseous treatment. (c) Quantification of [^3^H]‐thymidine incorporation in 14 DIV midbrain cultures receiving the same treatments as in (b). Data are means ± SEM (*n* = 15). *p <* *0.05 relative to control cultures under 75% N_2_. ^§^ *p *<* *0.05 relative to cultures maintained under 75% Xe and cultures maintained under 75% N_2_ and the same non‐gaseous treatment. (d) Quantification of [^3^H]‐thymidine incorporation in pure astrocyte cultures receiving the same treatments as in (b) and (c) for 3 DIV. Data are means ± SEM (*n* = 9). *p <* *0.05 relative to control cultures under 75% N_2_. ^§^ *p *<* *0.05 relative to cultures maintained under 75% Xe and cultures maintained under 75% N_2_ and the same non‐gaseous treatment.

This last set of observations suggested that GDNF, a peptide that exerts survival and trophic effects for midbrain DA neurons (Kramer and Liss 2015), was possibly involved in xenon's effects. At an optimal concentration of 20 ng/mL, GDNF provided the same level of protection as 75% xenon in this experimental setting (Fig. 6a). Yet, we found that an anti‐GDNF antibody (AB212 NA; 10 μg/mL) able to neutralize the protective effect of the trophic peptide, failed to reduce that of xenon (Fig. 6a). Importantly, the rescue provided by memantine, alone, or in combination with xenon remained also unaffected by the anti‐GDNF antibody.

### Neuroprotection of DA neurons by xenon results from the repression of a glial‐dependent mechanism

We next addressed the possibility that the neuroprotective action of xenon could result from the repression of a death mechanism mediated by glial cells, which proliferate spontaneously under present conditions. In support to this possibility, we showed that reducing glial cell proliferation with the antimitotic drug cytosine β‐d‐arabinoside (Ara‐C) (10 μM), led to a better survival of DA neurons in cultures that had been maintained under 75% N_2_ between 7 and 14 DIV More specifically, the survival of TH+ neurons was improved by 435% (*p* < 0.05) (Fig.  6b) and glial cell proliferation reduced by 53% (*p* < 0.05) (Fig. 6c) with such treatment. Both effects of Ara‐C appeared to be also improved by 75% xenon, although, not significantly.

Consistent with our working hypothesis, glial cell proliferation was slightly but significantly reduced (*p *<* *0.05) in midbrain cultures maintained under 75% xenon (Fig. 6c). Of interest, 10 μM memantine which strongly improved the rescuing effect of 75% xenon for DA neurons (Fig. 6b) also reinforced the anti‐proliferative action of the noble gas in these cultures (*p *<* *0.05) (Fig. 6c). Surprisingly, however, memantine exerted no repressive effect on glial cells in itself.

Astrocytes or their precursor cells which are the most abundant types of glial cells in midbrain cultures supplemented with serum (Mourlevat *et al*. 2003; Rousseau *et al*. 2013), represent a likely target for xenon‐mediated neuroprotection in the present setting. Thus, we carried out additional proliferation assays using astrocyte cultures. The anti‐proliferative effects of 75% xenon and that of 75% xenon combined to 10 μM memantine were also detected in these conditions. Ara‐C (10 μM) remained equally effective in both nitrogen and xenon atmospheres. Memantine, alone remained totally inactive in astrocyte cultures (Fig. 6d).

## Discussion

We demonstrate here that xenon but not argon provides partial but sustained protection to midbrain DA neurons in two distinct experimental settings that model the progressive loss of these neurons in PD. In both cases, the effect of xenon was mimicked and improved by the NMDA receptor antagonist memantine and the noble gas itself appeared to work by antagonizing NMDA glutamate receptors. Xenon exerted its protective action by means of either a direct or indirect effect onto DA neurons depending upon the experimental paradigm we used.

### Xenon protects DA neurons from sustained, low‐level excitotoxic stress induced by PDC

A number of reports suggest that DA cell death in PD may result from a slow excitotoxic process occurring as a consequence of an increased excitatory drive from subthalamic nucleus glutamatergic neurons (Blandini *et al*. 2001; Wallace *et al*. 2007; Michel *et al*. 2016). This process may result itself from reduced dopaminergic inhibitory control over subthalamic glutamatergic neurons (Piallat *et al*. 1999; Charles *et al*. 2008). The deficit in DA may also decrease the presynaptic inhibition that the neurotransmitter directly exerts onto DA neurons via D_2_ receptors, thus making these neurons further vulnerable to excitototoxic stress (Berman *et al*. 2011; Vaarmann *et al*. 2013).

To model a slow and sustained excitotoxic process as it may occur in PD, we exposed midbrain cultures to PDC, a structural analog of glutamate that does not act as an agonist of glutamate receptors on its own but does generate a toxic build‐up of the neurotransmitter in the extracellular milieu (Maki *et al*. 1994; Blitzblau *et al*. 1996). More precisely, PDC operates through inhibition of glutamate transport and stimulation of its release (Lavaur *et al*. 2016a). At 100 μM of PDC, a concentration killing about 75% of DA neurons over a period of 4 day, we noted that extracellular levels of glutamate were increased by two‐fold, confirming the view that a limited but sustained elevation of extracellular glutamate was potentially deleterious for DA neurons in this experimental setting (Nafia *et al*. 2008).

DA cell death induced by PDC was substantially reduced when the cell culture atmosphere was modified by replacing 75% nitrogen with 75% xenon. This observation is in line with previous works reporting on the neuroprotective potential of xenon in experimental paradigms that model acute neuronal insults in ischemic conditions (David *et al*. 2008; Thoresen *et al*. 2009; Banks *et al*. 2010; Deng *et al*. 2014). However, this is the first time that the protective effect of xenon is described in a paradigm that is relevant to PD‐induced neurodegeneration.

### Xenon does not interfere with the presynaptic action of PDC

In a model system of hypoxia‐induced neuronal damage, xenon was reported to be neuroprotective through its ability to reduce the release of glutamate in the extracellular milieu (Petzelt *et al*. 2003). This suggested that the neuroprotective effect of xenon for PDC‐treated DA neurons may possibly result from a presynaptic effect of the noble gas. This was unlikely as the increase in extracellular glutamate levels produced by PDC remained the same in cultures exposed to 75% xenon. This means that the noble gas worked post‐synaptically in the present setting through a direct effect onto DA neurons.

### The protective effect of xenon for PDC‐treated DA neurons is mediated by NMDA receptor blockade

Memantine, an antagonist of NMDA glutamate receptors was like xenon protective for DA neurons exposed to PDC, indicating that xenon may operate itself by antagonism of these receptors. Supporting this possibility, the noble gas remained strongly protective when NMDA, a prototypical agonist for NMDA receptors, replaced PDC to trigger DA neuron degeneration. Such a mechanism of action for xenon is also coherent with molecular dynamic stimulations that predict several putative binding sites for xenon on NMDA receptors (Liu *et al*. 2010). Besides, functional studies performed on cell lines overexpressing subunits of the NMDA receptor have shown that xenon has the capacity to reduce inward currents evoked by NMDA (Armstrong *et al*. 2012).

More specifically, xenon has been reported to compete with glycine, a positive allosteric modulator, at the NMDA receptor (Harris *et al*. 2013). Neurobasal medium used in the PDC paradigm contains a concentration of glycine that is saturating for the NMDA receptor glycine site, which indicates that xenon may be unable to compete efficiently with glycine under these conditions. This means that xenon may interfere with NMDA receptor function through another binding site, which has to be functionally characterized (Liu *et al*. 2010). In any case, the active binding site for xenon appears to be distinct from that of memantine (Lipton 2007; Kotermanski and Johnson 2009; Liu *et al*. 2010), which may explain why memantine enhanced the protective action of xenon for DA neurons.

Noticeably, another noble gas argon, which shares in common with xenon a filled valence shell, did not protect DA neurons from PDC. This means that the larger atomic radius of xenon (Zhang and Xu 1995) may be a key element in the ability of this noble gas to interfere with NMDA receptors and protect DA neurons.

### Xenon reduces the impact of oxidative stress‐mediated damage in PDC‐treated DA neurons

The overstimulation of NMDA receptors results in a series of downstream events, notably a rise in cytosolic calcium which stimulates in turn oxidative stress‐mediated neuronal damage (Brennan‐Minnella *et al*. 2013). In the present context, oxidative stress may also arise from the blockade by PDC of the excitatory amino acid carrier‐1 (Berman *et al*. 2011; Assous *et al*. 2014), a neuronal glutamate transport system that co‐transport cysteine, the rate‐limiting substrate for the synthesis of the major cellular antioxidant glutathione (Aoyama and Nakaki 2015).

Confirming that oxidative stress was involved in DA cell death induced by PDC, we observed that the free radical scavenger Trolox (Janc and Müller 2014) had the capacity to reduce DA cell demise when used at an optimal concentration of 10 μM. The use of the fluorogenic probe DHR‐123 revealed that oxidative stress returned to basal values in PDC‐treated neurons exposed to 75% xenon or to 10 μM Trolox, suggesting that xenon may possess intrinsic antioxidant properties. We have shown previously, however, that xenon was unlikely to operate as an antioxidant on its own (Lavaur *et al*. 2016a), which suggests that the impact of xenon on ROS production was indirect and solely due to its capacity to limit NMDA receptor overstimulation. Coherent with this view, oxidative stress induced by PDC was also inhibited by memantine, which like xenon, lacks antioxidant properties (Lavaur *et al*. 2016a).

Because Xenon provided only partial protection to PDC‐treated DA neurons, we asked whether Trolox had the capacity to improve this effect. We found that the protective effect of xenon was enhanced by concentrations of Trolox providing partial protection for DA neurons. Unexpectedly, however, concentrations of Trolox providing no protection at all against PDC, were able to substantially improve the protective action of 75% xenon. Studies performed with DHR‐123 revealed that low concentrations of Trolox that were not protective *per se* had, however, a small antioxidant effect which seemed sufficient to reinforce the efficacy of xenon against low and sustained excitotoxic insults. These observations may be particularly relevant to a disease state such as PD where oxidative stress appears crucially implicated (Jenner 2003; Salazar *et al*. 2008; Guerreiro *et al*. 2009).

### Xenon provides protection to spontaneously dying DA neurons

To further explore the neuroprotective potential of xenon for midbrain DA neurons, we used experimental conditions in which the loss of these neurons occurs spontaneously and progressively after plating (Wu *et al*. 2009). This paradigm is interesting in that it is even more progressive than the previous one. Besides, it allows for modeling a PD stage where DA cell loss has reached ~ 50% that is a point where clinical symptoms become apparent in patients (Ma *et al*. 1997; Damier *et al*. 1999; Jellinger 2012) and where neuroprotective treatments can be possibly administered to them.

Similar to what we observed in the previous paradigm, we found that xenon was partially protective for spontaneously dying DA neurons whereas argon remained totally ineffective. As Argon is strongly neuroprotective against acute ischemic insults (Brücken *et al*. 2013; Zhao *et al*. 2016), one may assume that its lack of protective effect in the present setting and also in the previous one was because of the slowly evolving nature of the insults in these two conditions.

Memantine like xenon afforded partial protection to spontaneously dying DA neurons, which is not totally unexpected if we refer to the work from Wu *et al*. (2009) showing that NMDA receptor antagonists promote DA cell survival in the same model system. Interestingly, we established that the protective effect of xenon was amplified by memantine, leading to the speculation that NMDA receptor antagonism was also, here, at the origin of the protective effect of the noble gas. A slow excitotoxic process was, however, unlikely to account for the spontaneous loss of DA neurons. Indeed, extracellular glutamate levels were only marginally increased during the time period during which treatments were applied to midbrain cultures (i.e., between 7 and 14 DIV) and these concentrations remained below 1.4 μM, i.e., well under the excitotoxic threshold. This also probably explains why the antioxidant Trolox, which provided partial protection against PDC‐induced DA cell death, remained totally ineffective in the present paradigm. Therefore, to explicate the protective action of xenon for spontaneously dying neurons, we explored other possible mechanisms.

### GDNF does not contribute to the protective effects of xenon for spontaneously dying DA neurons

The two NMDA receptor antagonists ifenprodil and memantine have been reported to stimulate the synthesis of the trophic factor GDNF in astrocytes (Toyomoto *et al*. 2005; Wu *et al*. 2009). Therefore, we studied the possibility that GDNF could operate as a mediator of xenon's effects in the present setting. Similar to what GDNF does (Kramer and Liss 2015), xenon provided both protection and trophic support to DA neurons. Yet, the protective effect of xenon was resistant to a neutralizing antibody (Toulorge *et al*. 2011) that prevented DA cell rescue provided by exogenous GDNF, which indicates that this trophic factor was most probably not involved in the effects of the noble gas. Note that the same antibody also failed to significantly reduce the protective effect of memantine alone or in combination with xenon. In line with these observations, GDNF remained undetectable in the culture medium of midbrain cultures treated with xenon (not shown).

### Xenon protects spontaneously dying DA neurons through a repressive effect on astroglial cells

Spontaneously dying DA neurons were maintained in the presence of serum proteins, a condition of culture reported to render astrocytes and their precursor cells potentially harmful for DA neurons (Mourlevat *et al*. 2003; Toulorge *et al*. 2010).This was probably the case, here, as reducing glial cell proliferation with the antimitotic Ara‐C, provided some protection to DA neurons. Thus, we hypothesized that the rescue by xenon was possibly the result of a repressive effect of the noble gas toward astroglial cells. Xenon had a modest inhibitory effect on the proliferation of glial cells in midbrain cultures and this effect was slightly more pronounced in pure astrocytic cultures, which is also consistent with a previous report showing that xenon can slightly delay cell cycle progression in astrocytic cells (Petzelt *et al*. 1999). Memantine which was more potent than xenon in protecting DA neurons in this model system exerted, however, no anti‐proliferative effect *per se* in midbrain cultures, which is somehow surprising as MK‐801, another NMDA receptor blocker was reported to reduce glioma cell proliferation (Ramaswamy *et al*. 2014). A possible effect of memantine on astrocyte function with no impact on cell proliferation is not totally excluded but remains to be proven. The anti‐proliferative effect of xenon toward glial cells was, however, enhanced by memantine in both midbrain and astrocytes cultures. This signifies that the level of protection provided by xenon, alone or xenon together with memantine was possibly related to the capacity of the two treatments to reduce the proliferation of astrocytes or their precursor cells, presumably through blockade of functional NMDA receptors on these cells (Verkhratsky and Kirchhoff 2007; Jimenez‐Blasco *et al*. 2015). Even if it remains to be demonstrated how glial and neuronal events are mechanistically interrelated, present data can be placed in perspective with other findings showing that reactive astrocytes may be key actors of neuronal death in a variety of degenerative conditions, including PD (Rousseau *et al*. 2013; Liddelow *et al*. 2017). Notably, soluble toxins or extracellular matrix components secreted by proliferating astroglial cells were reported to be permissive for DA cell demise (Rousseau *et al*. 2013; Liddelow *et al*. 2017).

In this setting, we also found that DA neurons were trophically and functionally stimulated by xenon, associated or not with memantine. This observation is reminiscent of earlier studies reporting that the repressive action of the antimitotic Ara‐C toward astroglial cells, resulted not only in better survival but also better differentiation of DA neurons in midbrain cultures (Mourlevat *et al*. 2003; Michel *et al*. 2013). This means that the small effects of xenon‐based treatments on proliferative astrocytes may be at the origin of both protective and trophic effects elicited by such treatments, even if we cannot totally exclude other possible mechanisms of action for xenon in the present model system.

Altogether, present data demonstrate that the noble gas xenon has the ability to provide protection and to exert trophic or restorative effects for DA neurons that are vulnerable in PD. Xenon had the potential to operate through a direct effect onto DA neurons and also through indirect mechanisms that probably implicate astrocytes. On the basis of these results, it is tempting to speculate about the therapeutic value of xenon in the pathology.
